# The Impact of Weight Bias and Stigma on the 24 h Dietary Recall Process in Adults with Overweight and Obesity: A Pilot Study

**DOI:** 10.3390/nu16020191

**Published:** 2024-01-06

**Authors:** Erica M. Howes, Molly K. Parker, Sarah A. Misyak, Alexandra G. DiFeliceantonio, Brenda M. Davy, Letisha Engracia Cardoso Brown, Valisa E. Hedrick

**Affiliations:** 1 Department of Human Nutrition, Foods and Exercise, Virginia Tech, Blacksburg, VA 24061, USA; pmolly95@vt.edu (M.K.P.); smisyak@vt.edu (S.A.M.); dife@vt.edu (A.G.D.); bdavy@vt.edu (B.M.D.); vhedrick@vt.edu (V.E.H.); 2 Fralin Biomedical Research Institute at VTC, Virginia Tech, Roanoke, VA 24016, USA; 3 Department of Sociology, University of Cincinnati, Cincinnati, OH 45221, USA; brown5lh@ucmail.uc.edu

**Keywords:** dietary assessment, obesity, weight stigma, 24 h recalls, indirect calorimetry

## Abstract

People with overweight and obesity tend to both underreport dietary energy intake and experience weight stigma. This exploratory pilot study aimed to determine the relationship between weight bias and weight stigma and energy intake reporting accuracy. Thirty-nine weight-stable adults with BMI ≥ 25 completed three 24 h dietary recalls; indirect calorimetry to measure resting metabolic rate; a survey measuring weight stigma, psychosocial constructs, and physical activity; and a semi-structured qualitative interview. Multiple linear regression was used to determine if weight bias internalization, weight bias toward others, and experiences of weight stigma were predictive of the accuracy of energy reporting. A thematic analysis was conducted for the qualitative interviews. Weight stigma was reported by 64.1% of the sample. Weight stigma constructs did not predict the accuracy of energy intake reporting. People with obesity underreported by a mean of 477 kcals (*p* = 0.02). People classified as overweight overreported by a mean of 144 kcals, but this was not significant (*p* = 0.18). Participants reported a desire to report accurate data despite concerns about reporting socially undesirable foods. Future research should quantify the impact of weight stigma on energy reporting in 24 h recalls using a larger, more diverse sample size and objective measures like doubly labeled water for validation.

## 1. Introduction

Weight bias and weight stigma are issues increasingly recognized by the medical and scientific communities [1] and have been shown to influence both psychological [2] and physical health [3]. The phrase weight bias describes negative stereotypes being applied to people with obesity [4]. Examples of these stereotypes include the assumption that individuals with obesity are inherently lazy, prone to overeating, or unable to make health behavior changes [1]. Weight stigma is the devaluation of people due to their body shape or weight [4]. Examples of weight stigma can include social exclusion or rejection, such as weight-based bullying or teasing [5]. One prominent place in which weight stigma is experienced is health care, where people with overweight or obesity may encounter judgmental comments, poor communication, or overall inferior care [6] as a result of interaction with health care providers [7]. Weight bias internalization, or internalized weight stigma, is the adoption of negative stereotypes and assumptions about people with obesity by people with higher weights [8]. Internalized weight bias has been shown to produce negative health consequences [9], both physically and mentally. Anticipated weight stigma, or the expectation that weight stigma will occur based on situational cues and past experiences with weight stigma [10], is another potential mechanism by which weight stigma can lead to poor health [11]. Finding ways to minimize the impacts of weight bias, weight stigma, and weight bias internalization is a priority for health research.

Underreporting of dietary energy intake is a phenomenon that has been consistently observed in nutrition studies, particularly among people with overweight and obesity [12]. Developing and refining methods of dietary assessment that promote valid estimates of habitual dietary energy intake is crucial for determining the relationship between diet and health, both in epidemiological research and in clinical studies [13]. As noted by Taylor et al. [14], it is vital to interpret the suitability and examine the validity of a dietary assessment tool for use in a given population. For example, the Automated Multiple Pass Method developed for use in the National Health and Nutrition Examination Survey has been shown to be valid among adults with normal weight but comparatively less valid among adults with overweight and obesity [15]. Despite the concerns about people with overweight and obesity underreporting their energy intake, the number of people with obesity in dietary validation studies is often limited [16]. As overweight and obesity prevalence continues to increase within the U.S. population [17], failing to recruit and enroll participants from all weight classes in representative levels limits the generalizability of validity results to populations with obesity.

Though it is well-documented that underreporting is observed more frequently and more severely among people with overweight and obesity [12,16], the etiology of the underreporting is still largely unknown [18]. Several psychosocial factors have been associated with underreporting, including social desirability [19,20,21,22], history of dieting [21,22,23,24,25,26,27,28,29], and reactivity to dietary assessment measures [30,31]. Given that individuals who regularly experience weight stigma are also more likely to exhibit body dissatisfaction [32,33], weight cycling [34], and disordered eating behaviors [3], studying whether weight stigma and the interrelationship of these variables contributes to underreporting of dietary energy intake is warranted.

To mitigate the possible negative impact of discrimination due to weight bias and stigma, participants in research studies may underreport their dietary energy intake. This behavior may be in response to weight-based social identity threat, in which weight stigma is anticipated based on certain environmental or social cues [11]. Therefore, underreporting may be more prevalent among people with higher body weights who are more likely to encounter weight stigma, particularly from health care professionals [35]. Given that clinical health research may be conducted in a setting similar to a health care setting, and by researchers who may be dietitians or otherwise involved in health care, it is unclear if negative experiences with weight stigma in health care or other settings could lead to anticipated weight stigma, negatively affecting research participants with overweight and obesity.

This study had three main objectives. The first objective was to determine the relationship between weight bias and weight stigma and energy intake reporting in adults with overweight and obesity in a research setting. We hypothesized that greater weight bias internalization among participants, past experiences of weight stigma among participants, and more weight bias toward people with obesity among participants would be associated with a greater degree of underreporting of dietary energy intake. The second objective was to explore what factors may contribute to the accuracy of dietary energy intake reporting and how weight stigma may impact participant experiences of the 24 h dietary recall process. Finally, the third objective was to determine the feasibility of recruiting individuals with overweight and obesity for a dietary assessment study to guide future research projects in this area. The second and third objectives were exploratory in nature and not hypothesis-driven.

## 2. Materials and Methods

### 2.1. Research Design

**Figure 1:**
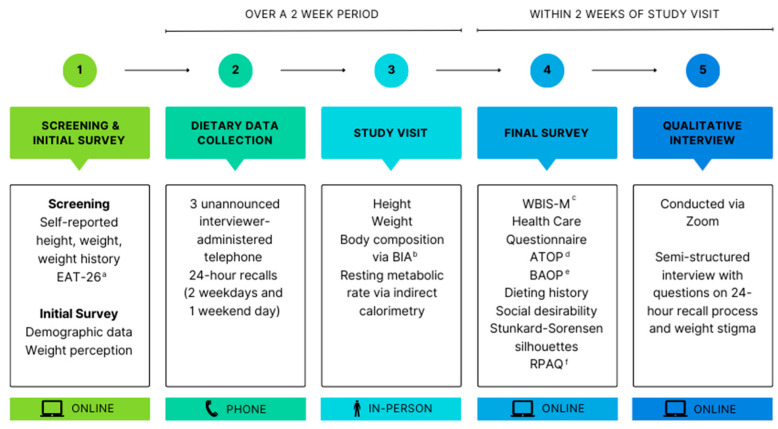
Study flow diagram for investigation of the impact of weight bias and stigma on energy misreporting in 24 h dietary recalls. ^a^ Eating Attitudes Test. ^b^ Bioelectrical impedance analysis. ^c^ Modified Weight Bias Internalization Scale. ^d^ Attitudes Toward Obese Persons Scale. ^e^ Beliefs About Obese Persons Scale. ^f^ Recent Physical Activity Questionnaire.

This exploratory pilot study used a mixed-methods, explanatory sequential design to examine the relationship between variables related to weight bias and energy intake reporting (Figure 1). The Virginia Tech Institutional Review Board approved the study protocol and all participants provided written informed consent.

### 2.2. Participants

A convenience sample of 39 adults with overweight and obesity living in or near a university community in the Southeast United States was recruited. Given the existing literature and exploratory nature of this study, a target sample size of approximately n = 40–50 was projected as sufficient to assess the objectives of this study [21]. Recruitment was completed through electronic mailing lists, social media platforms, and research institute websites. The sample was limited to adults with overweight and obesity due to the relatively high prevalence of weight bias internalization and weight stigmatizing experiences among adults with overweight and obesity [36].

### 2.3. Screening and Eligibility Criteria

Participants were screened via an online survey [37] assessing study eligibility. Inclusion criteria included adults ≥18 years old with a body mass index (BMI) ≥25 kg/m^2^ who were able to read and understand English and willing and able to complete all study activities. Exclusion criteria included a significant weight change of ≥4.5 kg in the previous two months [20], currently following a weight control diet, currently pregnant or breastfeeding, and/or screening positive for an eating disorder based on the Eating Attitudes Test-26 (EAT-26) questionnaire [38]. Participants scoring greater than 20 on the EAT-26; reporting any binge eating, vomiting, laxative use, diuretic use, or diet pill use; or reporting a loss of >20 pounds over the past six months were excluded from the study and provided with a list of treatment providers specializing in eating disorders and other local mental health resources via email.

Eligible participants completed the informed consent form and a demographic survey, which included age, sex assigned at birth, gender identity, educational attainment, race, ethnicity, and weight perception. Weight perception was assessed via one question asking “How would you describe your weight?” using a 7-point Likert scale with response options ranging from “extremely underweight” to “extremely overweight” [8].

### 2.4. Dietary Intake Assessment

Dietary energy intake was assessed via three unannounced telephone-administered 24 h recalls using a multiple-pass method. Data were collected for two nonconsecutive weekdays and one weekend day [39]. One graduate-level registered dietitian nutritionist (RDN), trained by a PhD-level RDN, performed the 24 h recalls to maintain consistency in recall collection. Dietary intake data were analyzed using Nutrition Data System for Research software 2021, developed by the Nutrition Coordinating Center, University of Minnesota, Minneapolis, MN, USA [40]. All dietary recalls were taken prior to any measurements of weight or weight stigma, to avoid a potential priming effect of weight stigma.

### 2.5. Study Visit

The study visit took place at a research institute. Participants were instructed to fast for eight hours, abstain from physical activity for 12 h, and abstain from stimulants, including caffeine or nicotine, on the morning of the visit [41]. Height in centimeters was measured using a wall-mounted stadiometer. Weight and body composition (percent body fat, fat mass, fat free mass, total body water) was measured using a Tanita TBF-310 bioelectrical impedance analysis scale. Participants were given the option to be weighed blindly if desired.

Indirect calorimetry was completed with a Parvo Medics TrueOne 2400 metabolic cart (Parvo Medics, Salt Lake City, UT, USA). Participants were seated in a reclining chair and asked to rest quietly in a dimly lit room with ambient temperature for 30 min prior to beginning the test. RMR was measured over a period of 30 min, taking measurements every minute. The first five minutes of the measurement were discarded to allow the participants to reach a steady state. Steady state was defined as a consecutive five-minute period in which participants had relatively stable levels of VO_2_ and VCO_2_, as defined by a coefficient of variation of ≤10% for each, and a respiratory quotient (RQ) within physiological norms (>0.7 and <1.0) [42]. When participants achieved steady state for more than one five-minute interval, the interval with the lowest coefficient of variation for VO_2_ and VCO_2_ was selected. The Weir equation was used within the Parvo Medics software (https://www.parvo.com/trueone-2400/, accessed on 29 October 2023) to calculate the RMR (in kcals/day) of participants [41]. The mean RMR for the five-minute interval selected was used as the assessment of the participant’s RMR [42,43].

### 2.6. Survey Measures

All participants completed a final study survey using paper and pencil or QuestionPro, an online survey platform [37]. Participants’ weight bias toward themselves was assessed through the Modified Weight Bias Internalization Scale [44]. Previous experiences of weight stigma in the healthcare setting were assessed using the “Health Care Questionnaire” [45]. For this study, the questions were modified to ask about health care providers in general, rather than only physicians. Participants’ weight bias toward others was assessed by the Attitudes Toward Obese Persons scale (ATOP) and the Beliefs About Obese Persons scale (BAOP) [46]. Given the importance of language in discussing different weight statuses [47,48] and the impact of language on weight stigma, the wording of the scale items was adjusted to use people-first language (i.e., people with obesity rather than obese people) [49].

Dieting history was measured using the history of dieting subsection of the Dieting and Weight History Questionnaire [50], which consists of four questions about the frequency and extent of previous dieting behaviors. The Marlowe–Crowne social desirability scale was used to assess participant social desirability [51]. The Stunkard–Sorensen silhouettes [52] were used to assess perception of body size, a measurement of body dissatisfaction. These silhouettes depict various body sizes of men and women using line drawings with numbers ranging from one, the thinnest figure, to nine, the largest figure. Participants were asked to select their ideal figure, the figure that reflects how they think they look, and the figure that reflects how they feel most of the time [53]. Participants’ body dissatisfaction was determined three ways based on the discrepancies between how participants feel minus what they selected as their ideal body shape, how they think they look minus what they selected as their ideal body shape, and how they feel minus what they think they look like [53].

Physical activity level for the previous four weeks was assessed using the Recent Physical Activity Questionnaire (RPAQ) [54]. The RPAQ is a self-reported questionnaire that captures information about each participant’s activities over the past 4 weeks in the domains of home activities (including sedentary time), commuting behaviors, work activities, and leisure time physical activity. It has been validated using doubly labeled water as a measure of physical activity energy expenditure [54]. The RPAQ was analyzed using the procedure described by Besson et al. and the procedure used in the National Diet and Nutrition Survey to obtain physical activity energy expenditure for each participant [55].

Participants were also asked to provide brief answers to three open-ended questions about their experiences completing the 24 h recall process, based on the questions used in the study by Vuckovic et al. [56]. The questions asked participants to describe their experience completing the 24 h recall generally, any difficulties completing the 24 h recall, and whether they felt their 24 h recall was representative of their true intake and why.

### 2.7. Qualitative Interview

Within 2 weeks of the study visit, all participants participated in one semi-structured qualitative interview with the same RDN who conducted the 24 h recalls. Interviews were conducted via Zoom [57], which was used to record audio and generate an initial transcript of each interview. Each transcript was then reviewed manually and edited as necessary to add contextual details or correct errors in the automatic transcription.

The interview guide for this study was developed collaboratively with the research team based on the specific aim of the qualitative portion of the study and with consideration of participants’ early responses to open-ended questions about the experience of completing the 24 h recall on the survey completed at the study visit. The initial interview questions were about the 24 h recall experience and followed a similar structure to the survey questions. Participants were also asked about any experiences with weight stigma and if applicable, efforts made to cope with weight stigma. For participants reporting any experiences of weight stigma, a final question was asked about any perceived relationship between weight stigma experiences and their experience with the 24 h recalls. Additional probes were determined by the interviewer as needed to provide additional richness to the participant responses.

### 2.8. Data Analysis

Quantitative data were analyzed using RStudio Version 2022.12.0+353. Scores were tabulated for each measurement of weight bias according to the scoring instructions [44,45,46]. Descriptive statistics were calculated for all measures collected. For continuous variables, *t*-tests were used exploratorily to determine if there were significant differences between participants with obesity and participants with normal weight or overweight. A Bonferroni correction was used to account for multiple comparisons. Participants who answered “prefer not to answer” for any questions in questionnaires where scores were computed were listed as “missing” for that questionnaire. The normality of all continuous variables was checked visually and using the Shapiro–Wilk normality test, with significance set at *p* < 0.05.

Accuracy of reporting was identified through a comparison between self-reported energy intake (EI) and estimated total energy expenditure (TEE). EI was calculated by averaging the total caloric intake estimated from the three 24 h recalls. TEE was calculated as the sum of the RMR from indirect calorimetry, physical activity energy expenditure as measured by the RPAQ, and the estimated thermic effect of food (10% of the sum of the previous two measurements) [20]. The accuracy of self-reported energy intake was expressed as a ratio of EI:TEE. Accuracy of reporting for the entire sample was computed using a Wilcoxon signed-ranks test [21]. Energy underreporters were classified as having an EI:TEE that fell below the 95% confidence limit of the log(EI:TEE), assuming a log(EI:TEE) of zero as perfect reporting, as described by Tooze et al. [22]. A Bland–Altman plot was used to express the agreement between EI and TEE. The effect of obesity status on energy reporting was determined using a linear mixed model. The R package lme4 was used, with REML and nloptwrap optimizer used to fit the model. A paired *t*-test was then used to determine if there were significant differences in energy reporting between participants with BMI < 30 and BMI ≥ 30.

Multiple linear regression analysis was used to determine the relationship between scores on the ATOP, BAOP, and Weight Bias Internalization Scale, experiences of weight stigma (yes/no), and EI:TEE [58]. Each of the variables of interest was evaluated individually, using EI:TEE as the dependent variable. Then, models were created that controlled for age, sex, percent body fat, level of education, social desirability scores, body dissatisfaction, and history of dieting. Only participants with complete data for all measures used in the models were included. All variables were checked for multicollinearity throughout the analysis process using variance inflation factors.

For the qualitative arm of the study, a thematic analysis was conducted. The interview transcripts were coded manually by two independent researchers. An initial codebook was developed based on review of a preliminary number of transcripts and modified to narrow the number of codes used. The two researchers independently reviewed the codes to determine the appropriateness of the coding scheme, and any new proposed codes were discussed and agreed upon before adding them to the codebook. The final codes were discussed before tabulating frequencies for each code and the percentage of participants reporting each code.

The portion of the interview that addressed the participant experience with the 24 h recall process was formally coded using a codebook. The portion of the interview that addressed weight stigma was coded using content analysis for each participant to determine (1) if they reported any experiences of weight stigma and (2) the type of weight stigma reported. These data were reported descriptively for the entire sample.

## 3. Results

A total of 39 eligible participants completed all study measures. Three people self-reported a BMI > 25, but they were excluded when their BMI was measured as <25 at the study visit. A total of five individuals dropped out prior to completing all study measures and were subsequently excluded from the final analysis. Of these individuals, two completed one 24 h recall, one completed two 24 h recalls, one completed three 24 h recalls, and one completed all study activities except for the final survey and qualitative interview. Final participant characteristics are described in Table 1. Participants ranged in age from 18 to 69 years old, with an average age of 43.3 years (SD = 15.0), and as a whole, they were highly educated, with 69.2% of participants holding a bachelor’s degree or higher. The majority of the participants (84.6%) were white. Over half (51.3%) of participants were classified as overweight. The mean BMI of all participants was 31.0 kg/m^2^ (SD = 6.47), and the mean body fat percentage for participants was 37.6% (SD = 8.43).

A summary of the psychosocial measures is provided in Table 2. This sample exhibited moderate levels of weight bias internalization, with a mean score of 3.54 (SD = 1.37) out of a possible score of 7. Participants with obesity had significantly higher (*p* = 0.003) levels of internalized weight bias (mean score 4.20) than participants with overweight (mean score 2.95). The mean score for the entire sample on the ATOP was 67.0 (SD = 18.9) out of a maximum of 120, with higher scores indicating more biased attitudes about people with obesity. The mean score on the BAOP was 20.9 (SD = 7.88) out of 48, with higher scores indicating more agreement with the belief that obesity is not under personal control. The mean body dissatisfaction for the entire sample was classified as low to moderate, with values of 2.42 (SD = 1.55) and 2.11 (SD = 1.16), for the discrepancy between participants’ selected ideal body shape and how they feel that they look (“feel” measure) and think that they look (“think” measure), respectively. Scores for the “think” measure of body dissatisfaction were significantly higher (*p* < 0.001) in participants with obesity (2.72, SD 0.895) compared to participants with overweight (1.55, SD 1.10). The sample had average scores on the Marlowe–Crowne social desirability scale, with a mean value of 16.0 (SD = 5.23) out of a possible score of 33. Of the 39 participants, 23 reported they had previously been on a diet to lose weight.

Much of the sample perceived that health care providers held negative attitudes or beliefs toward people with obesity (Table 3). Many participants reported being told by a health care professional to lose weight without asking. Specifically, 20.5% of the sample reported this experience happened “usually”, 20.5% reported it happened “sometimes”, and another 17.9% reported it happened “rarely”.

The majority (n = 25, 64.1%) of the participants reported an experience of weight stigma in the qualitative interview. The specific weight stigma experiences reported varied from isolated incidents in childhood to recurring, regular sources of stigma. One participant, a 52-year-old woman, reported the following in reference to weight stigma: “*It’s- sort of everywhere. It’s embedded in our social consciousness, so even if people aren’t intentionally going after it, it uh, shows up a lot of the time…”*. Other participants reported feeling a strong impact of weight stigma experiences as children or young adults but noted that aging changed how they viewed those experiences. A 56-year-old female participant stated “*Although at this point, I’m much more even keel about it probably- it probably has to do with age, I really think that has to do with age and experience…”*.

Of the 25 participants who reported previous experiences of weight stigma, 6 (24%) reported those experiences affected the way that they viewed or experienced the 24 h recall process. Most participants who experienced weight stigma (56%) stated they did not feel their experiences of weight stigma affected their experience with the 24 h recalls, and another 20% were unsure. However, some participants did note that the recalls brought up feelings about their body weight. For example, a 48-year-old female participant stated “*Um I- I mean, the recall just made me realize how [explicit] I eat. Which in turn, makes me feel [explicit] about my weight. Um, because it demonstrates how poorly- how poor my diet is”*. Another participant, a 19-year-old woman, noted that her body size led her to feel more worried about judgment when discussing food with another person: “*I feel like, as somebody who is overweight, I’m- just in general, I’m more likely to be judged for whatever I’m eating…”*.

**Figure 2:**
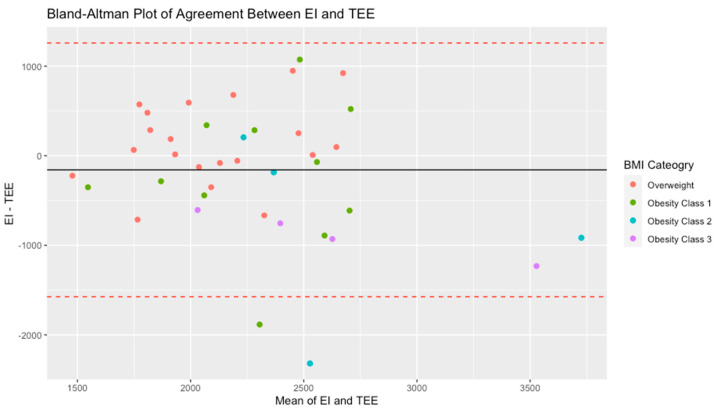
Bland–Altman plot of the agreement between energy intake (EI) in kcals as measured by 24 h recalls and total energy expenditure (TEE) in kcals, as measured by a sum of the resting metabolic rate (RMR), the thermic effect of food (TEF), and physical activity energy expenditure (PAEE) in a sample of 39 participants with overweight and obesity. Note: red dashed line represents the 95% confidence interval of the difference between EI and TEE.

There was not a significant difference between the EI and TEE for the entire sample (*p* = 0.32). Out of the 39 participants, 37 were classified as adequate energy reporters, and two were classified as energy intake underreporters. The Bland–Altman plot for the sample describes the agreement between EI and TEE (Figure 2).

**Figure 3:**
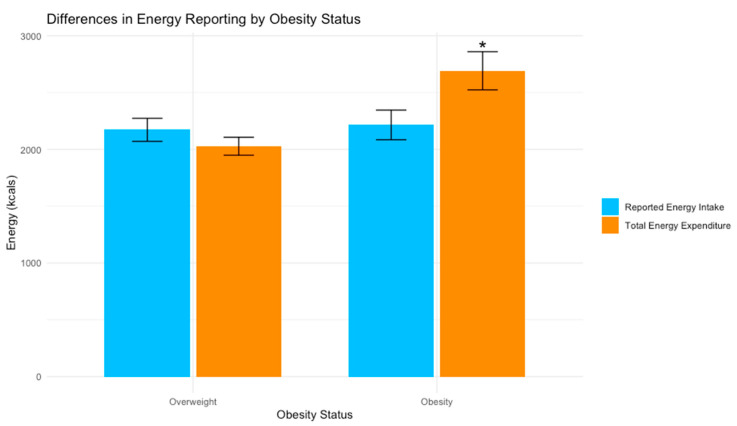
Differences in energy reporting by obesity status in a sample of 39 participants with overweight and obesity. Note: Error bars represent ±1 SE. Reported energy intake measured by 24 h recalls; energy expenditure measured by a sum of the resting metabolic rate (RMR), the thermic effect of food (TEF), and physical activity energy expenditure (PAEE). * significant difference between reported energy intake and total energy expenditure (*p* < 0.05).

The linear mixed model indicated substantial explanatory power with a conditional R^2^ of 0.32. When predicting the total energy from the energy type (intake or expenditure) and obesity status (BMI ≥ 30 or <30), there was a significant negative effect of the interaction of the energy type and obesity status on the total energy (F = 8.73, *p* = 0.004). A paired *t*-test indicated significant underreporting (mean difference = −477 kcals, *p* = 0.02) among people with obesity (BMI ≥ 30), while people without obesity (BMI < 30) did not exhibit significant underreporting (mean difference = 144 kcals, *p* = 0.18) (Figure 3).

The results of the multiple regression analysis indicated that experiences of weight stigma or scores on the Weight Bias Internalization Scale, ATOP, or BAOP were not significant predictors of the degree of energy intake reporting (EI:TEE). These variables were not significant when added to the linear model individually, or when controlling for age, sex, body fat percentage, educational level, social desirability, body dissatisfaction, and dieting history (Table 4).

The thematic analysis produced several major themes relating to the question of why and in what ways participants may underreport dietary energy intake related to their experience of weight stigma (Table 5). Many participants reported sentiments consistent with social desirability. One 52-year-old woman stated “*I’m a people pleaser, so I think having somebody having to tell somebody that I ate a slice of carrot cake three nights in a row [laughs] um, kind of made me think of myself as, ‘Oh, you probably shouldn’t have done that.’ You know what I mean, like it’s- it’s the recall of saying ‘oh I ate that’”*.

A second prominent theme was increased awareness of food choices around the study period. Many people reported thinking more about their food choices or reflecting more on their food choices after reporting them in the 24 h recalls. According to a 27-year-old female participant, “*And so it was an interesting experience to just sort of be consciously aware of the food choices that I was making because I usually just don’t even think about it”*.

However, few participants reported that these feelings of hesitation or embarrassment about reporting their food choices or awareness of their food intake led to a decrease in food intake during the study period. A third theme was “no changes to intake” or maintaining normal eating habits throughout the study. One 56-year-old participant stated “*You know, I think I thought sometimes, ‘Oh, maybe I should make sure I do this a little better,’ but I don’t think I really changed anything, no. No. [laughs] I really didn’t. And then that- the morning I’d say, ‘Oh there’s a recall today. Oh well, that’s what I ate yesterday that’s what it is’ [laughs]”*.

Many participants also noted a desire to make dietary changes, such as reducing calories or making changes to the types of foods consumed, but reported barriers to engaging in those behavior changes, such as busy schedules, typical dietary patterns, or enhanced sensitivity to food cues in their environments. One 29-year-old female participant stated the following:

“*So, it made me want to make behavioral changes. But I think it was still difficult to do, just because it was such a busy time, work-wise and in my life. So um, and it’s yeah, I just I think it made behavioral changes for like, the next day, but not long term, cause it was like, I noticed it, made a quick change, but then I remembered how crazy life was, and like, why I didn’t have time to cook the day before, and then I was back to ordering out.*”

A major motivator for participants to report their foods accurately throughout the research process was respect for the research process. Many commented on their desire to provide accurate data for the study or expressed concern that they were not providing data that were accurate enough. This also tended to be a reason why participants did not alter their normal eating habits during the study, as many were thinking about the need to provide representative data. Participants often noted using strategies to enhance food recall, such as writing out all the foods they consumed the previous day in the morning before speaking with the interviewer and adding foods to the list as they recalled them. One 21-year-old female participant expressed feeling tempted to report in a socially desirable manner thinking about the intention of the research study to collect data led them to challenge that thought: “*I’m like, ‘Oh, like I should be super healthy, and she’s going to be like, so proud of me, for, like all the things I ate,’ and the other part of me is like, ‘this research study is not to like judge, like- like it doesn’t matter how well I do, like that’s not what this is about.’”* Another participant, a 47-year-old woman, reported noticing that emotions came up during the dietary recall process, and it took conscious effort to resist “messing up” the data by reporting in a different way: “*I was trying to be aware of that and I knew that I had to be brave to make the study accurate, because I mean, we’re trying to get [laughs] data points here and make science happen and we can’t let all the fuzzy emotions, you know, mess that up so*”.

A final major theme reported about the 24 h recall experience was a lack of judgment. Many people specifically mentioned an expectation to feel judged during the research process, but in contrast, ultimately feeling a lack of judgment during the research process. A 56-year-old female participant stated “*You know, cause you think, you know, because not that anyone is judging you, but there is a feeling that people might judge you*”.

## 4. Discussion

Weight stigma is a common experience among people with overweight and obesity. In this sample, over half of participants reported an experience of weight stigma in their lives, including within the healthcare setting. Internalized weight stigma, experiences of weight stigma, and attitudes and beliefs toward people with obesity were not significantly predictive of the degree of underreporting of energy intake in this study. For the entire sample, there were no significant differences between reported energy intake and estimated daily energy expenditure. However, when examining energy reporting by obesity status, participants with obesity exhibited significant underreporting, while participants with overweight did not exhibit significant underreporting.

The greater degree of energy underreporting found among participants with obesity is consistent with previous findings [16]. People with obesity reported similar levels of calories in the 24 h recalls as people with overweight, but had significantly greater energy expenditure, indicating greater underreporting of energy intake. While this study used self-reported physical activity, of which overreporting in participants with obesity may have contributed to the higher levels of energy expenditure found in this group, the overall trends are consistent with the findings of Lissner et al. [59] and Moshfegh et al. [15], who both found greater underreporting among people with obesity compared to people with normal weight or overweight. Similarly, Waterworth et al. [60] found that people with obesity and people without obesity reported similar amounts of calories in a four-day food record, but the people with obesity had much higher energy expenditures according to DLW measurements, resulting in higher absolute underreporting. They proposed an allometric scaling method that would account for the higher energy expenditures found with higher body masses. In Waterworth’s study, using this allometric scaling method removed the effect of obesity on underreporting [60].

One potential reason for the relatively low overall rates of underreporting found in this study was the effort by many participants to provide accurate data. This sample was primarily drawn from a university community; thus, the sample was highly educated, with many having a strong motivation to provide accurate data to advance research. Though participants were not informed that the study would be validating their reported energy intake through measures of energy expenditure, the collection of objective measures such as indirect calorimetry as part of the study may have also implicitly incentivized accurate reporting if participants perceived these measures as able to verify their self-reported dietary data. Though participants noted some perceived discomfort or embarrassment with reporting less socially desirable foods such as desserts or restaurant foods, the desire to provide accurate data for research seemed to outweigh the fear of judgment. Few participants reported experiencing reactivity to the measure by decreasing their dietary intake during the study. Most participants stated they made no changes to their typical dietary intake due to the recalls being unannounced and thus being unable to manipulate their diet for the entire study period. Some noted that they had the thought of wanting to make dietary changes because of their enhanced awareness of their dietary choices, but instead defaulted to their normal dietary patterns.

In addition, this study was designed with a concerted effort to minimize possible weight stigma, which may have produced lower rates of underreporting compared to other similar studies. This study used person-first language throughout study materials, and recruitment materials were kept neutral, advertising the study as being about “dietary assessment and weight”. Participants were not informed about the aims of the study until all study measures had been completed. The 24 h recalls were also conducted via phone, prior to any measurement of participants’ body weights, which may have mitigated participant worries about how the interviewer perceived their body weight or potential for weight stigma. This would be consistent with the observations of Blodorn et al., who found that among women with higher body weights, worrying about social rejection increased when potential dating partners could view information about their body weight as compared to when no body weight information was visible [61].

Body dissatisfaction is also an important factor to consider related to underreporting. Novotny et al. [21] found a link between a desire to weigh less and underreporting of dietary energy intake in two 24 h recalls. Kagawa and Hills [62] similarly found a link between body dissatisfaction and energy intake underreporting, though the study primarily studied individuals classified as normal weight. In a study of Greek adults, Kanellakis et al. [63] found greater odds of underreporting in 24 h recalls for individuals with a negative perception of their body image, particularly among women. In this study, there was significantly more body dissatisfaction among participants with obesity compared to participants with normal weight or overweight, which is consistent with previous research [64]. The underreporting found among people with obesity, but not among people with no obesity, suggests body dissatisfaction remains an important factor to consider in future studies of people with obesity.

Though there was no link found between weight stigma-related variables and underreporting in this study, the high prevalence (64.1%) of weight stigma is relevant to future studies on overweight and obesity. This level of weight stigma is consistent with the prevalence of weight stigma reported by Lessard et al. [65]. Future studies examining dietary intake or weight that include people with overweight and obesity should consider the potential impact of weight stigma on prospective participants. Though weight stigma was not predictive of underreporting in this study, participants did report feeling social embarrassment or discomfort with discussing their food intake, which in some cases related to feelings about their body weight. Dietary studies seeking to recruit participants with overweight and obesity should consider these potential feelings of embarrassment or fear of judgment, which may inhibit recruitment and retention of individuals with higher weights. Examples of strategies that may mitigate potential feelings of weight stigma during research procedures include using people-first language [49] when discussing weight in study recruitment materials, asking about participant preferences when taking weight measurements, and avoiding the perpetuation of stereotypes about obesity in images and communications (e.g., using images of people eating “unhealthy” foods in study recruitment materials) [66,67].

This study had several limitations that provide insight into results and can help guide the development of future research. The use of a convenience sample resulted in a highly educated group of participants with little racial or ethnic diversity. Additionally, many participants were classified as overweight, which may have limited the prevalence of weight stigma within this sample, as higher levels of weight stigma are observed in participants in higher weight classes [68]. Future work should stratify recruitment to ensure that there is adequate representation of participants from all weight classes and strategically target recruitment to increase sample diversity. Additionally, experiences of weight stigma were measured dichotomously (yes or no) and did not take into account frequency or duration of weight stigma experiences, factors which could affect the potential impacts of the stigmatizing experiences. Finally, while the qualitative methods can suggest relationships between variables to help explain the findings, establishing definitive relationships between variables would require future studies using validated methods to measure each construct of interest.

The method used to determine TEE relied partially on self-reported information about physical activity, which may be subject to potential reporting biases in a similar way to self-reported dietary intake [69]. An objective measure of physical activity, such as accelerometry, would provide a way to validate participants’ self-reported physical activity levels; however, this approach is costly. In addition to the use of accelerometry, future work may consider using a recovery biomarker such as DLW [12], which takes into account the total energy expenditure, including physical activity expenditure, and could be used as a proxy for dietary energy intake among weight-stable participants to identify underreporting.

## 5. Conclusions

This study represents an innovative approach to dietary assessment work by bridging the areas of weight stigma and dietary assessment validation. The qualitative findings demonstrating the prevalence of weight stigma among adults with overweight and obesity underscore the importance of considering weight stigma in dietary studies. Though no associations were found between weight stigma and underreporting of dietary energy intake in this study, many participants reported feeling worried that the dietary data collection process might lead to judgment from researchers or increased feelings of self-judgment about food and weight. Future studies using larger, more diverse sample sizes and improved validation methods are needed to more comprehensively explore the relationship between weight stigma and dietary reporting.

## Figures and Tables

**Table 1:** Participant characteristics (n = 39) for the total sample and by obesity status.

Characteristic	Overall (n = 39)	%	Overweight (n = 20)	%	Obesity (n = 19)	%
Sex						
Male	6	15.4	3	15.0	3	15.8
Female	33	84.6	17	85.0	16	84.2
Gender identity						
Man	7	17.9	3	15.0	4	21.1
Woman	32	82.1	17	85.0	15	78.9
Age (y) mean (SD)	43.3 (15.0)		38.8 (15.1)		48.1 (13.8)	
Educational attainment						
High school	8	20.5	2	10.0	6	31.6
Associate’s degree	4	10.3	3	15.0	1	5.3
Bachelor’s degree	11	28.2	7	35.0	4	21.1
Master’s degree	11	28.2	4	20.0	7	36.8
Doctoral degree	5	12.8	4	20.0	1	5.3
Ethnicity						
Hispanic/Latino	2	5.1	2	10.0	0	0
Not Hispanic/Latino	37	94.9	18	90.0	19	100
Race						
Asian	3	7.7	2	10.0	1	5.3
Black	1	2.6	1	5.0	0	0
White	33	84.6	15	75.0	18	94.7
More than one race	2	5.1	2	10.0	0	0
Body Mass Index (kg/m^2^) mean (SD)	31.0 (6.47)		26.7 (1.61)		35.5 (6.66)	
Percentage body fat (BIA ^a^) mean (SD)	37.6 (8.43)		32.2 (5.93)		43.3 (6.81)	
Weight Perception						
Normal weight	7	17.9	7	35.0	0	0
Slightly overweight	10	25.6	9	45.0	1	5.3
Overweight	16	41.0	4	20.0	12	63.2
Extremely overweight	6	15.4	0	0	6	31.6

^a^ BIA: bioelectrical impedance analysis.

**Table 2:** Psychosocial measures (n = 39) for the sample and by obesity status.

	Overall (n = 39)	Overweight (n = 20)	Obesity (n = 19)	*p*-Value ^a^
Weight Bias Internalization ^b^				
Mean (SD)	3.54 (1.37)	2.95 (1.48)	4.20 (0.867)	0.003 *
Missing ^c^ (n)	1	0	1	
Social Desirability ^d^				
Mean (SD)	16.0 (5.23)	16.1 (6.46)	15.8 (3.94)	0.874
Missing (n)	6	4	2	
ATOP ^e^				
Mean (SD)	67.0 (18.9)	72.5 (16.7)	61.8 (20.0)	0.103
Missing (n)	6	4	2	
BAOP ^f^				
Mean (SD)	20.9 (7.88)	22.8 (8.16)	18.9 (7.27)	0.134
Missing (n)	2	1	1	
Body Dissatisfaction ^g^ (Feel—Ideal)				
Mean (SD)	2.42 (1.55)	1.80 (1.47)	3.11 (1.37)	0.007 *
Missing (n)	1	0	1	
Body Dissatisfaction ^h^ (Think—Ideal)				
Mean (SD)	2.11 (1.16)	1.55 (1.10)	2.72 (0.895)	<0.001 *
Missing (n)	1	0	1	

^a^ *p*-value from independent-samples *t*-test comparing BMI < 30 with BMI ≥ 30 for continuous variables. The Bonferroni correction was used to account for multiple comparisons with a significance level of *p* = 0.008. ^b^ Weight Bias Internalization was measured using the Modified Weight Bias Internalization Scale [44]. Scores range from 1 to 7, with higher scores indicating more internalized weight bias. ^c^ Responses coded as “missing” included participants who selected “prefer not to answer” or did not complete all questions for a given questionnaire. ^d^ Social Desirability was measured using the Marlowe–Crowne Social Desirability Scale [51]. Scores range from 0 to 33, with higher scores indicating more social desirability. ^e^ ATOP: Attitudes Toward Obese Persons Scale [46]. Scores range from 0 to 120, with higher scores indicating more positive attitudes toward people with obesity. ^f^ BAOP: Beliefs About Obese Persons Scale [46]. Scores range from 0 to 48, with higher scores indicating greater belief that the causes of obesity are not personally controllable. ^g^ Measured using the Stunkard–Sørensen silhouettes [52], and for each person, taking the difference between the figure that best represents how they feel minus their ideal figure. Lower scores represent lower body dissatisfaction. ^h^ Measured using the Stunkard–Sørensen silhouettes [52], and for each person, taking the difference between the figure that best represents what they think they look like minus their ideal figure. Lower scores represent lower body dissatisfaction. * *p* < 0.001, significant using Bonferroni-corrected *p*-value of 0.008.

**Table 3:** Summary of responses to the Health Care Questionnaire ^a^ about negative experiences related to weight (n = 39).

	n (%)		n (%)
When I lost weight and regained it, health care professionals criticized me for not trying harder.		I feel that I cannot speak freely with health care professionals about my weight.	
Never	26 (66.7%)	Never	20 (51.3%)
Rarely	5 (12.8%)	Rarely	9 (23.1%)
Sometimes	3 (7.7%)	Sometimes	5 (12.8%)
Usually	2 (5.1%)	Usually	4 (10.3%)
Always	1 (2.6%)	Always	0 (0%)
Missing	2 (5.1%)	Missing	1 (2.6%)
Health care professionals have said critical or insulting things to me about my weight.		I feel that health care professionals don’t believe me when I tell them that I don’t eat that much.	
Never	20 (51.3%)	Never	22 (56.4%)
Rarely	9 (23.1%)	Rarely	7 (17.9%)
Sometimes	8 (20.5%)	Sometimes	5 (12.8%)
Usually	1 (2.6%)	Usually	3 (7.7%)
Always	0 (0%)	Always	0 (0%)
Missing	1 (2.6%)	Missing	2 (5.1%)
I have been very upset by comments that health care professionals have made about my weight		I feel that health care professionals don’t treat overweight people as nicely as they do average-weight people.	
Never	20 (51.3%)	Never	7 (17.9%)
Rarely	11 (28.2%)	Rarely	7 (17.9%)
Sometimes	6 (15.4%)	Sometimes	15 (38.5%)
Usually	1 (2.6%)	Usually	9 (23.1%)
Always	0 (0%)	Always	0 (0%)
Missing	1 (2.6%)	Missing	1 (2.6%)
I feel that I have been treated disrespectfully by people in the health care profession because of my weight.		Health care professionals have told me that I need to lose weight without my asking them.	
Never	26 (66.7%)	Never	15 (38.5%)
Rarely	6 (15.4%)	Rarely	7 (17.9%)
Sometimes	4 (10.3%)	Sometimes	8 (20.5%)
Usually	2 (5.1%)	Usually	8 (20.5%)
Always	0 (0%)	Always	0 (0%)
Missing	1 (2.6%)	Missing	1 (2.6%)
Health care professionals have tried to scare me into losing weight by warning me about health risks associated with being overweight.		I feel that most health care professionals don’t understand how difficult it is to be overweight.	
Never	20 (51.3%)	Never	7 (17.9%)
Rarely	9 (23.1%)	Rarely	8 (20.5%)
Sometimes	5 (12.8%)	Sometimes	8 (20.5%)
Usually	4 (10.3%)	Usually	13 (33.3%)
Always	0 (0%)	Always	1 (2.6%)
Missing	1 (2.6%)	Missing	2 (5.1%)

^a^ The Health Care Questionnaire [45] was developed to capture perceptions of health care among people with obesity and includes a subsection on negative experiences related to weight with physicians. This study modified the term “physician” to “health care provider” to capture attitudes related to a broader group of health care providers.

**Table 4:** Prediction of the ratio of reported energy intake (EI) to total energy expenditure (TEE) in a sample of adults with overweight and obesity (n = 39) ^a^.

Model	Prediction Variables	*R* ^2^	*β*	Significance (*p*-Value)
1a	WBIS-M ^b^	0.02	−0.22	0.18
1b	WBIS-M	0.09	−0.46	0.32
2a	Weight stigma (yes vs. no) ^c^	−0.02	−0.09	0.59
2b	Weight stigma (yes vs. no)	−0.03	−0.09	0.53
3a	ATOP ^d^	−0.03	0.04	0.83
3b	ATOP	−0.16	0.15	0.75
4a	BAOP ^e^	0.01	0.18	0.28
4b	BAOP	−0.10	0.23	0.68

^a^ Each model used the ratio of reported energy intake (EI) to total energy expenditure (TEE) as the dependent variable. The “a” models are unadjusted linear models, and the “b” models are adjusted for age, sex, body fat percentage, educational level, social desirability, history of dieting, and body dissatisfaction. ^b^ WBIS-M: Modified Weight Bias Internalization Scale [44]. ^c^ Self-reported history of experiencing weight stigma, collected via qualitative interview. ^d^ ATOP: Attitudes Toward Obese Persons Scale [46]. ^e^ BAOP: Beliefs About Obese Persons Scale [46].

**Table 5:** Themes, frequencies, and representative quotes derived from qualitative interviews regarding the experience of completing 24 h recalls among participants with overweight and obesity (n = 39).

Code	Description	% of Participants Reporting	Representative Quote
No changes to intake	No reported changes to intake during study period	74	“I don’t actually think it changed- like change my behavior. I know I wasn’t eating differently, because of it, I just ate what I wanted to eat, and then I tried to best of my ability actually tell you what it was.”—41-year-old man
Social desirability	Guilt about acceptability of food choices; desire to make a favorable impression on interviewer	69	“I think- it- the idea of talking to a stranger about what you’ve eaten and not knowing what their reaction is to it, right, having it be, whether it’s over video or over phone, like, what are they thinking of me?”—55-year-old man
Awareness	Increased awareness of intake	67	“So I think I started to be a little bit more conscious of what I was eating, or just like more aware. I didn’t feel negative about it. I think I just- noticed how much I was not cooking [laughs] um, as I was doing the recalls.” —29-year-old woman
Empirical nature of research	Desire for accuracy, justifying accurate reporting due to nature of research	54	“But, the science part of it’s like, well, if I didn’t tell you what I was eating, then you wouldn’t know what I was eating, and that would affect the study. Do you know what I mean?” —47-year-old woman
Neutral/positive recalls	No negative impact of recalls	41	“Um, I did, like during the actual phone call, it was pretty neutral every single time.” —19-year-old woman
No judgment	Expectations or perceptions of judgment about food intake during recall	41	“I like, didn’t worry about you necessarily judging me because I was like this, like I didn’t feel like you were going to.”—21-year-old woman
Dieting	Mention of intentional dieting, tracking, or weight loss program	26	“I would say, from a memory perspective, I didn’t find it hard, but I’ve done so many diets where you track foods, so I think I’m pretty cognizant of what I’m putting in.” —46-year-old woman
Changes to intake during study	Restricting intake during study period	15	“Um. I- well, you know, I definitely decided I wasn’t going to binge eat chips [laughs] if I knew I was going to be talking to you, and then because I didn’t know when I was going to be talking to you, you know, I stayed away from things like that the entire week.”—60-year-old woman

## Data Availability

The data presented in this study are available on request from the corresponding author. The data are not publicly available due to participant privacy protections.
